# BCG arthritis in a patient with Severe Combined Immunodeficiency Disorder (SCID): a case report

**DOI:** 10.1186/s12887-026-06866-8

**Published:** 2026-04-22

**Authors:** Shabnam Eskandarzadeh, Zahra Chavoshzadeh, Mohammad Assadi Fanid, Babollah Ghasemi, Amirreza Jabbaripour Sarmadian

**Affiliations:** 1 Pediatric Health Research Center, Tabriz University of Medical Sciences, Tabriz, Iran; 2 Immunology and Allergy Department, Mofid Children’s Hospital, Shahid Beheshti University of Medical Sciences, Tehran, Islamic Republic of Iran; 3 Department of Orthopaedics, Zahra Mardani Azari Children Training, Research and Treatment Center, Tabriz University of Medical Sciences, Tabriz, Iran; 4 Division of Clinical Laboratory, Zahra Mardani Azari Children Training, Research and Treatment Center, Tabriz University of Medical Sciences, Tabriz, Iran; 5 Tabriz USERN Office, Universal Scientific Education and Research Network (USERN), Tabriz, Iran

**Keywords:** Severe combined immune deficiency, Bacillus Calmette Guerin vaccine, Tuberculosis vaccine, Arthritis, Case report

## Abstract

**Background:**

Severe combined immunodeficiency (SCID) is a rare group of primary immunodeficiency disorders characterized by profound defects primarily in T cells and, in certain subtypes, in B and NK cells as well. These immune deficiencies predispose affected patients to life-threatening infections and complicate their clinical management. Since the BCG vaccine is routinely administered to neonates in most countries, undiagnosed SCID patients face a significant risk of developing severe localized or disseminated BCG-related complications. Therefore, it should be taken into account in suspicious populations.

**Case presentation:**

The patient was a 16-month-old Iranian male with *JAK3 gene* mutation-associated SCID who presented with progressive left elbow swelling, pain, and restricted mobility over 10 days. Physical examination revealed tachycardia, fever, marked swelling and tenderness with severely restricted range of motion, and a firm, fixed, warm palpable mass. Soft tissue ultrasound demonstrated moderate joint effusion with synovial thickening, multiple irregular hypoechoic areas, and increased Doppler vascularity consistent with chronic monoarthritis and periarticular masses of probable bacterial origin. Given the lack of response to empirical therapy and conservative management, surgical excision was performed. Histopathological examination revealed neoplastic spindle cell proliferation with hyperchromatic nuclei, epithelioid macrophages, mixed inflammatory infiltrate, and numerous acid-fast bacilli both intracellularly and extracellularly. To reliably distinguish BCG from other members of the Mycobacterium tuberculosis complex, molecular analysis was performed, which demonstrated deletion of the RD1 region, confirming BCG arthritis. Following surgery and antimycobacterial therapy, the patient demonstrated marked clinical improvement, particularly in elbow range of motion, and was discharged after completing intravenous antibiotics and achieving clinical stabilization.

**Conclusions:**

This case underscores the critical need for revised immunization policies in TB-endemic countries, implementation of systematic neonatal primary immunodeficiency screening programs such as TREC screening, and enhanced healthcare provider awareness regarding the potentially catastrophic consequences of BCG vaccination in immunocompromised populations.

## Background

Severe combined immunodeficiency (SCID) represents a rare and heterogeneous spectrum of primary immunodeficiency disorders (PIDs) resulting from underlying genetic defects that critically impair the development and functional capacity of essential immune cell populations. Although T lymphocytes constitute the primary cellular target across all SCID variants, the pathological process extends to encompass B lymphocytes and natural killer (NK) cells in specific disease subtypes, thereby compromising multiple arms of the adaptive and innate immune response [1, 2]. These profound immunological impairments predispose affected individuals to life-threatening complications, most notably severe opportunistic infections and a markedly elevated risk of mortality [3]. The severity of these immune defects necessitates specialized care and intensive medical monitoring, yet the clinical management of SCID patients remains challenging due to the complexity and heterogeneity of their underlying immunological deficiencies [4, 5].

A particularly noteworthy clinical challenge involves the administration of the Bacillus Calmette-Guérin (BCG) vaccine [6, 7], which is routinely administered to newborns in approximately 156 countries worldwide [8]. Given that SCID often remains clinically undiagnosed during the neonatal period, a substantial proportion of affected patients receive BCG vaccination before their immune disorder becomes apparent, potentially leading to severe BCG-related complications that range from localized and regional manifestations to distant and disseminated disease [9]. The clinical spectrum of these complications typically includes respiratory symptoms, vaccination site inflammation, lymphadenopathy, cutaneous eruptions, and fever, while arthritis represent rare but documented complications [10, 11]. We present herein the case of a 16-month-old patient with SCID and prior BCG vaccination who presented with swelling, pain, and restricted mobility of the left elbow.

## Case presentation

The patient was a 16-month-old Iranian male toddler who was brought to the outpatient clinic by his parents with complaints of swelling, pain, and restricted mobility in the left elbow, symptoms which had commenced approximately 10 days prior and had progressively deteriorated since onset. Born to consanguineous parents as their first child, the patient’s medical history was notable for complications that ultimately led to his SCID diagnosis.

The patient’s clinical course began with hospitalization at 5 months of age for infectious gastroenteritis, followed by the discovery of severe lymphopenia at 6 months. SCID was suspected based on a reduced T-cell receptor excision circle (TREC) count (1512 copies per 10⁶ cells), which was below the laboratory reference range; the normal range for the patient’s age was ≥ 5862 copies per 10⁶ cells. Therefore, flow cytometry was performed, revealing an immunophenotype consistent with leaky SCID. The diagnosis was subsequently confirmed by whole-exome sequencing (WES), which identified a pathogenic *Janus Kinase 3 (JAK3) gene* mutation. Following this diagnosis, the patient was initiated on monthly intravenous immunoglobulin (IVIG) therapy along with prophylactic antimicrobial medications. It should be noted that, prior to diagnosis, the patient had received all vaccinations according to the national immunization schedule. Specifically, the BCG vaccine was administered at birth, delivered intradermally into the upper left arm (deltoid region) in accordance with standard immunization protocol.

Then, after diagnosis, vaccination was continued in accordance with the recommended specialized immunization protocol for patients with immunodeficiency, as outlined by the Advisory Committee on Immunization Practices (ACIP), European Society for Immunodeficiencies (ESID), Infectious Diseases Society of America (IDSA), and the World Health Organization (WHO), which includes the use of inactivated vaccines and the avoidance of live vaccines [12, 13]. Of particular clinical significance, the patient had been considered a candidate for hematopoietic stem cell transplantation (HSCT); however, this treatment could not be performed due to donor-related issues. Despite good adherence to the treatment regimen, he experienced a significant clinical setback at 9 months of age, requiring hospitalization for pneumonia. Notably, two months prior to the current presentation, at 14 months of age, he had experienced mild, self-limiting swelling in the left knee, which may have represented an early manifestation of his current underlying condition.

Physical examination revealed concerning vital signs, including a blood pressure of 100/65 mmHg, tachycardia with a heart rate of 152 bpm, fever with an axial body temperature of 38.4 °C, a respiratory rate of 36 breaths per minute, and oxygen saturation of 96% on room air. The left elbow demonstrated notable swelling, tenderness, and significantly restricted active and passive range of motion. Palpation revealed a firm, non-mobile, and warm mass.

The clinical findings raised concerns for a possible underlying soft tissue mass, most likely of infectious origin; therefore, the patient was admitted under strict isolation precautions for comprehensive evaluation. Before initiating antibiotic therapy, blood, urine, cerebrospinal fluid (CSF), and stool cultures were obtained to thoroughly assess for potential bacterial infection. Then, IVIG, intravenous fluids, and high-dose empirical antibiotic and antifungal therapies were promptly administered while the patient’s condition was closely monitored, an approach deemed appropriate given the severity of his symptoms and the unavailability of culture results at the time.

To further characterize the swelling and restricted joint mobility, soft tissue ultrasound of the left elbow was performed, revealing moderate joint effusion accompanied by synovial thickening and multiple irregular hypoechoic soft tissue areas extending around the anterior aspect of the joint. Doppler imaging showed increased vascularity within the synovium, findings consistent with active inflammation and suggestive of chronic monoarthritis with concern for periarticular soft tissue masses of probable bacterial etiology.

**Fig. 1:**
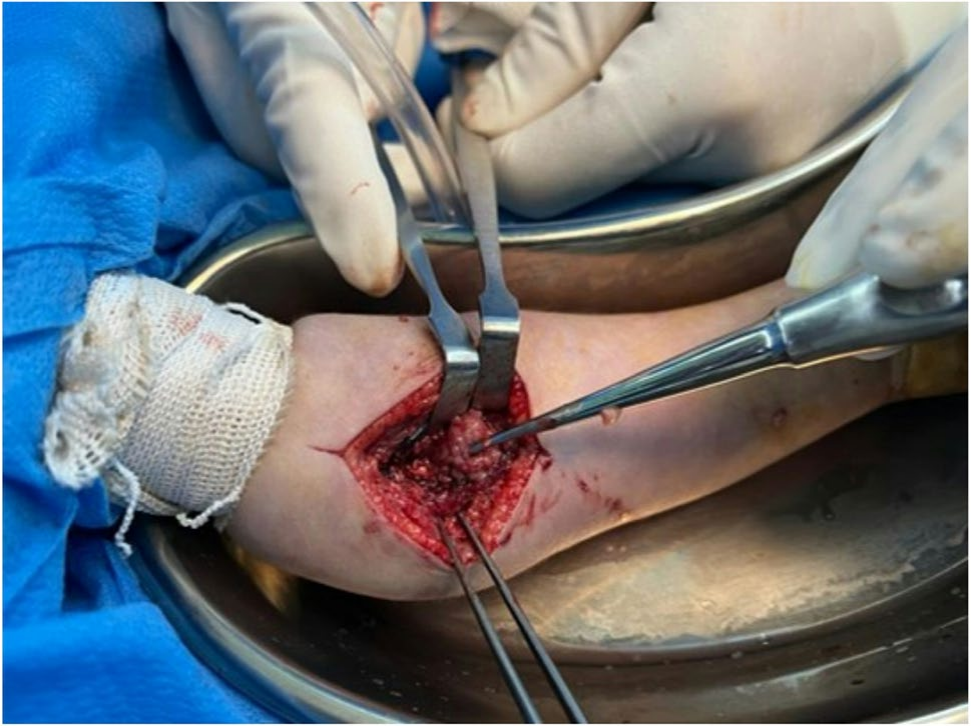
Intraoperative anterior view of the left elbow during surgical excision of hypervascular soft tissue masses

Despite three days of continued antibiotic therapy, the patient showed no significant clinical improvement. Furthermore, all initial culture results were reported as negative. Given the persistence of symptoms and lack of substantial response to conservative management, surgical intervention was undertaken to excise the masses for both diagnostic and therapeutic purposes, as shown in Fig. 1.

**Fig. 2:**
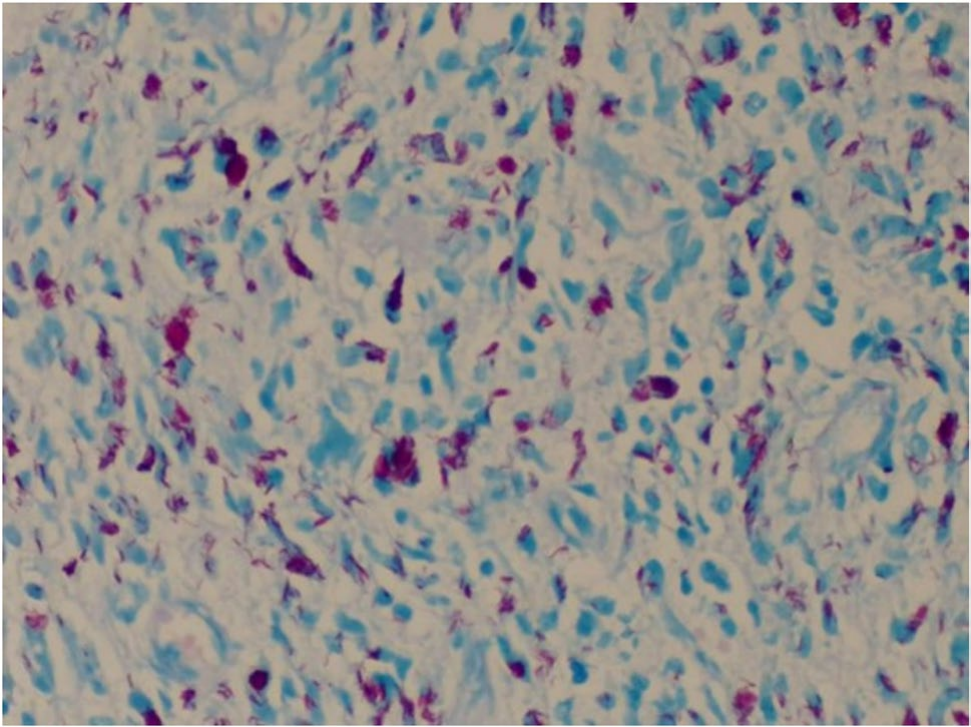
Ziehl-Neelsen stain of excised soft tissues showing numerous intracellular and extracellular acid-fast bacilli

Following surgical excision of the masses, specimens were submitted for comprehensive histopathological evaluation. Microscopic examination revealed neoplastic proliferation of plump spindle cells characterized by hyperchromatic vesicular nuclei and eosinophilic cytoplasm lacking distinct cell borders, accompanied by epithelioid macrophages and scattered mixed inflammatory cells. Significantly, numerous acid-fast bacilli were identified, predominantly within cells but also distributed in extracellular spaces. To reliably distinguish BCG from other members of the *Mycobacterium tuberculosis* complex, molecular analysis was performed, which demonstrated deletion of the RD1 region, a well-established genetic hallmark differentiating BCG strains. Based on the combined histopathological, microbiological, and molecular findings, a definitive diagnosis of BCG arthritis was established, as shown in Fig. 2.

In parallel, to further assess for infection beyond the left elbow, systemic imaging and organ-specific evaluations were performed. The only additional abnormality identified was within the urinary tract, where imaging findings revealed bladder infection, including the presence of a fungal ball. No radiologic or clinical signs of infection were detected in other organs, including the lungs, liver, spleen, or bone marrow.

After confirming the diagnosis, the patient was initiated on targeted broad-spectrum antimicrobial therapy, including rifampin, isoniazid, ethambutol, ciprofloxacin, and amikacin, as well as amphotericin B. Within several days of surgery and treatment initiation, he demonstrated marked clinical improvement, particularly with regard to the range of motion of the left elbow joint. Following completion of the intravenous antibiotic course and clinical stabilization, the patient was discharged.

Antimicrobial therapy was continued with oral administration, prescribed with strict adherence instructions. During regular follow-up visits over a one-year period, the patient was closely monitored for the emergence of additional complications. IVIG therapy was maintained throughout this period. However, the patient required multiple hospitalizations due to recurrent infections, none of which were related to BCG, and has not undergone HSCT yet.

## Discussion

In our case, the patient presented with left elbow swelling, fever, and tachycardia. Additionally, he had a history of unexplained left knee swelling that occurred two months prior to admission, which had resolved. This clinical presentation raised our initial suspicion of infectious etiology, as recurrent infections are a hallmark feature of PID patients [14]. The most important differential diagnosis in this patient was septic arthritis, if left untreated, it could rapidly lead to joint destruction, systemic infection, and even sepsis, particularly in immunocompromised individuals [15]. Other differential diagnoses include infectious monoarthritis, soft tissue infections (abscess, cellulitis), osteomyelitis, autoimmune inflammatory conditions, reactive arthritis, and neoplastic processes. Based on the ultrasound findings, the clinical suspicion of septic arthritis and infectious monoarthritis was further supported. Therefore, blood, urine, CSF, and stool cultures were obtained. Joint aspiration was not initially performed due to technical difficulties and the risk of exacerbating the patient’s symptoms, as the affected joint showed signs of severe inflammation and swelling. Subsequently, IVIG, intravenous fluids, and high-dose empirical antibiotic and antifungal therapies were promptly administered. Despite the persistence of symptoms and lack of substantial response to conservative management, and in parallel with the negative cultures, surgical intervention was undertaken. Combined histopathological, microbiological, and molecular findings ultimately led to a definitive diagnosis of BCG arthritis. This constellation of findings, including systemic symptoms such as fever and involvement of distant joints, strongly suggests that the patient developed disseminated BCG disease rather than a localized or regional infection. Notably, the prior episode of self-limiting knee swelling may represent a herald symptom and a missed opportunity for earlier recognition of disseminated disease. As discussed in previous studies, arthritis is one of the less commonly reported manifestations of this condition.

Regarding the medical treatment, after confirming the diagnosis, the patient was initiated on targeted antimycobacterial therapy, including rifampin, isoniazid, and ethambutol for BCG arthritis, alongside ciprofloxacin and amikacin as broad-spectrum antibiotics. Pyrazinamide was not included in the treatment regimen due to the intrinsic resistance of *Mycobacterium bovis* (including BCG strains) to this agent. The broad-spectrum antibiotics were administered because the patient had an underlying immune deficiency and had undergone surgery, both of which put them at increased risk for opportunistic infections. Additionally, amphotericin B was included due to the simultaneous presence of a fungal infection in the bladder, which required antifungal treatment to prevent further systemic dissemination.

The BCG vaccine represents one of the most extensively utilized immunization interventions in global public health history. It has been administered to over 4 billion children across more than 180 countries over nearly a century of clinical use. Currently, approximately 200 million doses are distributed annually worldwide [16, 17]. Originally derived from an attenuated strain of *Mycobacterium bovis* and first introduced in Paris in 1921, this vaccine subsequently underwent rigorous clinical evaluation and proved to be highly effective in protecting against primary tuberculosis (TB) over the following decades. Consequently, WHO officially incorporated it into its Expanded Programme on Immunization (EPI) in 1974, establishing it as a cornerstone of global pediatric immunization strategies and a key intervention in international TB control efforts [17].

National BCG vaccination policies have been tailored to reflect regional TB patterns and healthcare priorities across different countries. Nations experiencing high TB burden typically maintain comprehensive vaccination programs targeting entire populations, while regions with lower disease prevalence implement selective approaches focusing on high-risk groups [18, 19]. The updated third BCG World Atlas illustrates the widespread adoption of this intervention, revealing that the majority of countries (156 out of 194) maintain universal neonatal BCG vaccination programs, with 51 of these nations being classified as low TB burden countries [8]. Notably, despite nearly a century of medical advances and ongoing research into alternative approaches, BCG remains the only vaccine approved for clinical protection against TB, highlighting both its continued importance in global health policy and the ongoing need for careful considerations regarding the vaccine itself [20].

While adverse reactions following BCG vaccination are rare, the vaccine’s live attenuated nature requires careful precautions in immunocompromised patients. Particularly, individuals with PIDs are particularly susceptible to vaccine-related complications, as BCG is typically administered at birth, before the first clinical manifestations of their immune system deficiency [21]. Fekrvand et al. [6] conducted a systematic review of PIDs associated with adverse events following BCG vaccination and reported that SCID was not only the most frequently reported condition but also demonstrated the highest BCG-related mortality rate. Therefore, special attention and precautionary actions should be taken for these patients, as well as for other susceptible populations, to minimize the risk of adverse outcomes.

To date, more than 20 genes have been identified as responsible for SCID [22], including the *JAK3 gene* located on chromosome 19p13.11, where mutations lead to autosomal recessive *JAK3 deficiency* (OMIM: 600173). It accounts for approximately 7–14% of SCID cases, and represents the type found in our patient. In the mentioned systematic review, only four patients with *JAK3 deficiency* and BCG-related complications were reported, highlighting the rarity of this condition [6].

Marciano et al. [9] conducted a retrospective multicenter study from 28 centers in 17 countries on 349 SCID patients who received BCG vaccination and found that 51% developed adverse reactions, with 17% experiencing localized and 34% disseminated reactions. In patients with disseminated disease, the most common presentations involved extra-regional lymph nodes (57%), skin (56%), and lungs (47%), while liver, spleen, and bone involvement were less frequent (15%, 13%, and 13% respectively). BCG isolation from bone marrow occurred in 14% of disseminated cases, with positive blood cultures being rare (1%). They also documented 46 deaths related to BCG vaccine complications.

In another study, Botaro et al. [10] evaluated 11 SCID patients, of whom 8 received BCG vaccination. Among the vaccinated patients, 87% developed adverse reactions, with 14% experiencing localized and 86% disseminated reactions. The most frequent clinical manifestations were pulmonary involvement (wheezing, cough, dyspnea, and respiratory failure), which was the primary presentation in all patients, followed by extra-regional lymphadenopathy, hepatosplenomegaly, as well as renal, cutaneous, and meningeal involvement. BCG isolation was achieved in 71% of cases, all of which were disseminated infections. The sources of isolation included skin lesions, lungs, liver, spleen, lymph nodes, and stomach, with isolation confirmed through necropsy examination in two patients. The single patient with localized disease presented with regional suppurative lymphadenopathy.

Cocchi et al. [11] retrospectively examined the clinical course of BCG disease in 36 SCID patients who underwent HSCT, all of whom had documented BCG vaccination. Among these patients, 92% developed adverse reactions, with 30% experiencing localized and 70% disseminated reactions. The most frequent clinical manifestations included inflammation at the vaccination site, lymphadenopathy, skin rash, and fever, whereas central nervous system involvement and arthritis were rarely observed.

## Conclusion

In conclusion, in patients with immune deficiencies, such as those with SCID, who have received the BCG vaccine at birth, BCG arthritis should be considered if they present with joint symptoms, including progressive swelling, pain, and restricted mobility, along with fever and tachycardia. Early recognition of immunodeficiency in infants is critical for preventing severe complications associated with BCG vaccination. According to the study by Fekrvand et al. [6], Iran, China, and Turkey have the highest frequencies of PIDs with BCG-related adverse events, and our patient is from Iran. Their findings, along with our report, underscore the urgent need to revise immunization schedules in TB-endemic countries, including Iran, as well as implementing systemic neonatal PID screening programs such as TREC screening, and increasing awareness among primary healthcare providers and physicians about the potentially catastrophic consequences of BCG vaccination in vulnerable populations.

## Data Availability

The datasets used and/or analysed during the current study are available from the corresponding author on reasonable request.

## Abbreviations

ACIP: Advisory Committee on Immunization Practices
BCG: Bacillus Calmette-Guérin
CSF: Cerebrospinal Fluid
EPI: Expanded Programme on Immunization
ESID: European Society for Immunodeficiencies
HSCT: Hematopoietic Stem Cell Transplantation
IDSA: Infectious Diseases Society of America
IVIG: Intravenous Immunoglobulin
JAK3 gene: Janus Kinase 3 gene
NK cells: Natural Killer cells
PIDs: Primary Immunodeficiency Disorders
SCID: Severe Combined Immunodeficiency
TB: Tuberculosis
TREC: T-cell Receptor Excision Circle
WES: Whole-Exome Sequencing
WHO: World Health Organization
